# Proinflammatory Effect of High Glucose Concentrations on HMrSV5 Cells via the Autocrine Effect of HMGB1

**DOI:** 10.3389/fphys.2017.00762

**Published:** 2017-09-29

**Authors:** Yuening Chu, Yi Wang, Zhihuang Zheng, Yuli Lin, Rui He, Jun Liu, Xuguang Yang

**Affiliations:** ^1^Department of Nephrology, Shanghai General Hospital, School of Medicine, Shanghai Jiao Tong University, Shanghai, China; ^2^Department of Oncology, Renji Hospital, School of Medicine, Shanghai Cancer Institute, Shanghai Jiao Tong University, Shanghai, China; ^3^Department of Immunology and Key Laboratory of Medical Molecular Virology of Ministries of Education and Health, School of Basic Medical Sciences, Fudan University, Shanghai, China

**Keywords:** high glucose, HMrSV5 cell, HMGB1, inflammation, apoptosis, MAPKs

## Abstract

**Background:** Peritoneal fibrosis, in which inflammation and apoptosis play crucial pathogenic roles, is a severe complication associated with the treatment of kidney failure with peritoneal dialysis (PD) using a glucose-based dialysate. Mesothelial cells (MCs) take part in the inflammatory processes by producing various cytokines and chemokines, such as monocyte chemoattractant protein 1 (MCP-1) and interleukin 8 (IL-8). The apoptosis of MCs induced by high glucose levels also contributes to complications of PD. High mobility group protein B1 (HMGB1) is an inflammatory factor that has repeatedly been proven to be related to the occurrence of peritoneal dysfunction.

**Aim:** In this study, we aimed to explore the effect and underlying mechanism of endogenous HMGB1 in high-glucose-induced MC injury.

**Methods:** The human peritoneal MC line, HMrSV5 was cultured in high-glucose medium and incubated with recombinant HMGB1. Cellular expression of HMGB1 was blocked using HMGB1 small interfering RNA (siRNA). Apoptosis and production of inflammatory factors as well as the potential intermediary signaling pathways were examined.

**Results:** The major findings of these analyses were: (1) MCs secreted HMGB1 from the nucleus during exposure to high glucose levels; HMGB1 acted in an autocrine fashion on the MCs to promote the production of MCP-1 and IL-8; (2) HMGB1 had little effect on high-glucose-induced apoptosis of the MCs; and (3) HMGB1-mediated MCP-1 and IL-8 production depended on the activation of MAPK signaling pathways. In conclusion, endogenous HMGB1 plays an important role in the inflammatory reaction induced by high glucose on MCs via mitogen-activated protein kinase (MAPK) signaling pathways, but it seems to have little effect on high-glucose-induced apoptosis.

## Introduction

Peritoneal dialysis (PD) is a successful renal replacement therapy for end-stage renal disease (ESRD) that effectively extends the lives of patients. However, prolonged exposure to bio-incompatible dialysis solutions results in impairment of the peritoneal membrane (PM), leading to PM deterioration and the eventual discontinuation of PD (Devuyst et al., 2010). High glucose as a major component of peritoneal dialysates plays an important role in the structural and functional alteration of the peritoneum by inducing apoptosis, inflammation, and epithelial-to-mesenchymal transition (EMT) of peritoneal mesothelium (Yu et al., 2009; Hung et al., 2014).

Mesothelial cells (MCs) are a critical component of the PM. Previous studies have confirmed the vital role of MCs in alterations of the PM. During responses to a wide range of exogenous or endogenous stimuli, MCs actively participate in the induction of the inflammatory response by serving as important contributors of many cytokines, such as monocyte chemoattractant protein 1 (MCP-1) and interleukin 8 (IL-8), which cause intra-peritoneal recruitment of leukocytes (Strippoli et al., 2016). Apoptosis of MCs is also a key factor contributing to cell death and complications in PD (Hung et al., 2014).

High mobility group protein B1 (HMGB1) is a nuclear protein with dual functions. HMGB1 is routinely located in the nucleus, where it regulates DNA replication, transcription, recombination, and repair (Ueda and Yoshida, 2010; Malarkey and Churchill, 2012). HMGB1 also acts as a damage-associated molecular pattern (DAMP) molecule that meditates proinflammatory response upon secretion into the extracellular environment (Li et al., 2014). HMGB1 interacts with specific receptors, including receptor for advanced glycation end products (RAGE), toll-like receptor (TLR)-2 and TLR-4 (Tang et al., 2010; Chen et al., 2016). The binding of HMGB1 to its receptors to initiate pro-inflammatory responses and inhibit apoptosis has been fully demonstrated (Lotze and Tracey, 2005; Thorburn et al., 2009; Lee et al., 2012).

Serum levels of HMGB1 have been associated with micro-inflammatory states in continuous ambulatory peritoneal dialysis (CAPD) patients, and inhibition of HMGB1 has a protective effect on peritoneal function in experimental models of peritonitis (Zhu et al., 2011; Cao et al., 2013). However, the pathogenic role of HMGB1 in high-glucose-meditated induction of cytokines and apoptosis in MCs remains largely unexplored. Therefore, in this study, we aimed to explore the effects of HMGB1 on high-glucose-mediated MC injury and the underlying molecular mechanism.

## Materials and methods

### Cell lines

Human peritoneal MC line HMrSV5 was kindly provided by Professor Pierre Ronco, Hospital Tenon, Paris, France. The cells were cultured in Dulbecco's Modified Eagle Medium (DMEM; Gibco, NY, USA) containing 10% fetal bovine serum (FBS; Hyclone, Logan, USA) at 37°C in humidified 5% CO_2_. To mimic the high-dialysate glucose environment, DMEM were supplemented with 84, 138, and 236 mM glucose, which are equal to dianeal peritoneal solution containing 1.5, 2.5, and 4.25% glucose currently used for PD in China mainland. In addition, the control group (ctrl) was treated with normal glucose at 5.6 mM and mannitol was used as the osmotic control. Recombinant human HMGB1 (rHMGB1) (Sigma, St Louis, USA) was added to the culture medium of cells for real time PCR, Enzyme-linked immunosorbent assay (ELISA), western blot assay, and apoptosis assay.

### Flow cytometry for apoptosis assay

Apoptosis detection was performed with fluorescein isothyocyanate (FITC) Annexin V Apoptosis Detection Kit (BD Biosciences, San Jose, USA). Briefly, cells were collected and washed twice with ice-cold binding buffer and then re-suspended in 400 μL of binding buffer. Three microliters of Annexin V-FITC stock solution was added to the cells and incubated for 15 min at 4°C. The cells were then further incubated with 3 μL of propidium iodide for 5 min at 4°C. The stained samples were immediately analyzed by fluorescence-activated cell sorting (FACS) Cyan instrument. Annexin V-FITC-binding positive-staining cells were scored as apoptotic. Apoptotic cells were counted and represented as a percentage of the total cell count.

### RNA isolation and quantitative real-time PCR (qPCR)

Total RNA was extracted using TRIZOL (Invitrogen, Carlsbad, USA), and cDNA was generated using High-capacity cDNA Reverse Transcription kit (TaKaRa Bio, Shiga, Japan). Quantitative real-time PCR was performed using SYBR green Gene Expression Assay (TaKaRa Bio, Shiga, Japan). The primer sequences of all genes used for PCR are as follows: *HMGB1*, 5′-GCGAATTCTGGGCAAAGGAGATCCTAAGA-3′ and 5′-GCGGTACCCGCTAGAACCAACTTATTCATCATC3-3′; *IL-8*, 5′-ACTGAGAGTGATTGAGAGTGGAC-3′ and 5′-AACCCTCTGCACCCAGTTTTC-3′; *MCP-1*, 5′-TTCTGTGCCTGCTGCTCAT-3′ and 5′-GGGGCATTGATTGCATCT-3′; and *GAPDH: 5*′-GAGTCAACGGATTTGGTCGT-3′ and 5′-TGGAAGATGGTGATGGGATT-3′.

### Western blotting assay

Cells were harvested and lysed in radioimmunoprecipitation-assay buffer with phenylmethanesulfonyl fluoride. The protein concentration was quantified using a bicinchoninic acid (BCA) protein kit (Pierce, Rockford, IL, USA). A total of 15 μg of whole cellular lysates was electrophoresed in 10% sodium dodecyl sulfate-polyacrylamide gel electrophoresis (SDS-PAGE) and blotted on a polyvinylidene disfluoride (PVDF) membrane (Millipore, Billerica, USA). The membranes were blocked with 5% fat-free milk at room temperature for 1 h and then incubated with primary antibodies including anti-ERK1/2 phospho-specific antibody (1:1,000; Cell Signaling Technology, Danvers, USA), anti-p38 phospho-specific antibody (1:1,000; Cell Signaling Technology), anti-JNK phospho-specific antibody (1:1,000; Cell Signaling Technology, Danvers, USA), anti-HMGB1 (1:4,000; Abcam, Cambridge, USA) antibody, or anti-GAPDH (1:4000; Cell Signaling Technology, Danvers, USA) antibody at 4°C overnight. After five washes for 5 min in phosphate-buffered saline (PBS) supplemented with 0.1% Tween-20 (PBST), the membrane was incubated with horseradish peroxidase (HRP)-conjugated secondary antibody (1:4,000; Cell Signaling Technology, Danvers, USA) for 2 h at room temperature. Enhanced chemiluminescence kit (Pierce, Rockford, IL, USA) was used for detection.

### Immunofluorescence assay

Cells in logarithmic growth phase were cultured for 24 h, and then the culture medium was replaced with a medium containing 236 mM of glucose (high glucose medium) for 48 h. Cells were washed with PBS, fixed with methanol, and sealed with 1% bovine serum albumin (BSA) for 2 h. The cells were then incubated with anti-HMGB1 (1:400; Abcam, Cambridge, USA) overnight at 4°C. The cells were incubated with green fluorescent secondary antibody in a dark room for 60 min, followed by incubation with 4′,6-diamidino-2-phenylindole (DAPI) (1:10,000; sigma, St. Louis, USA) for 1 min. Fluorescent intensity was observed with an inverted fluorescence microscope.

### RNA interference

HMGB1 small interfering RNA (siRNA) was purchased from Biotend Co., Ltd. (Shanghai, China). Cells were seeded into 12-well-culture plates at a density of 2 × 10^5^ cells per well the day before transfection. HMGB1-targeted and negative control siRNA were dissolved separately in Opti-MEM. The solutions were equilibrated for 5 min at room temperature, and each RNA solution was combined with Lipofectamine 3000 reagent (Invitrogen, Carlsbad, USA). The samples were mixed gently and allowed to sit for 25 min to form siRNA liposomes. The HMrSV5 cells were transfected with the transfection mixture in antibiotic-free cell culture medium. The protein levels of HMGB1 were detected after 24 h of transfection. After 24 h of transfection, the medium was replaced with high glucose culture medium, and MCP-1 and IL-8 were detected after another 24 h.

### ELISA

Secretion of HMGB1 (Shino-Test, Kanagawa, Japan), tumor necrosis factor alpha (TNF-α), IL-8, and MCP-1 (Peprotech, Rocky Hill City, USA) were determined in culture supernatant of MCs using a sandwich ELISA. All assays were performed according to the manufacturer's instructions. Triplicate samples were analyzed using an ELISA reader and compared to a standard curve.

### Mitogen-activated protein kinase (MAPK) inhibitor treatment

SB203580, U0126, and SP600125 (Selleck, Houston, USA) were chosen to block the activation of p38, ERK, and JNK, respectively. The inhibitors were dissolved in dimethyl sulfoxide to make 10 mM stock solutions. Working concentrations of 0.5 μM SB203580, 0.07 μM U0126, and 90 nM SP600125 were used. Cells were pretreated with inhibitors for 30 min before incubation with rHMGB1.

### Statistical analysis

The comparisons between two groups were performed by two-tailed Student's *t*-tests. Multiple-group comparisons were performed by two-way analysis of variance (ANOVA). Statistical analysis was performed with GraphPad Prism 6 (GraphPad Software, Inc.). Significant difference was defined as *p* < 0.05.

## Results

### High glucose induces inflammation in MCs

**Figure 1:**
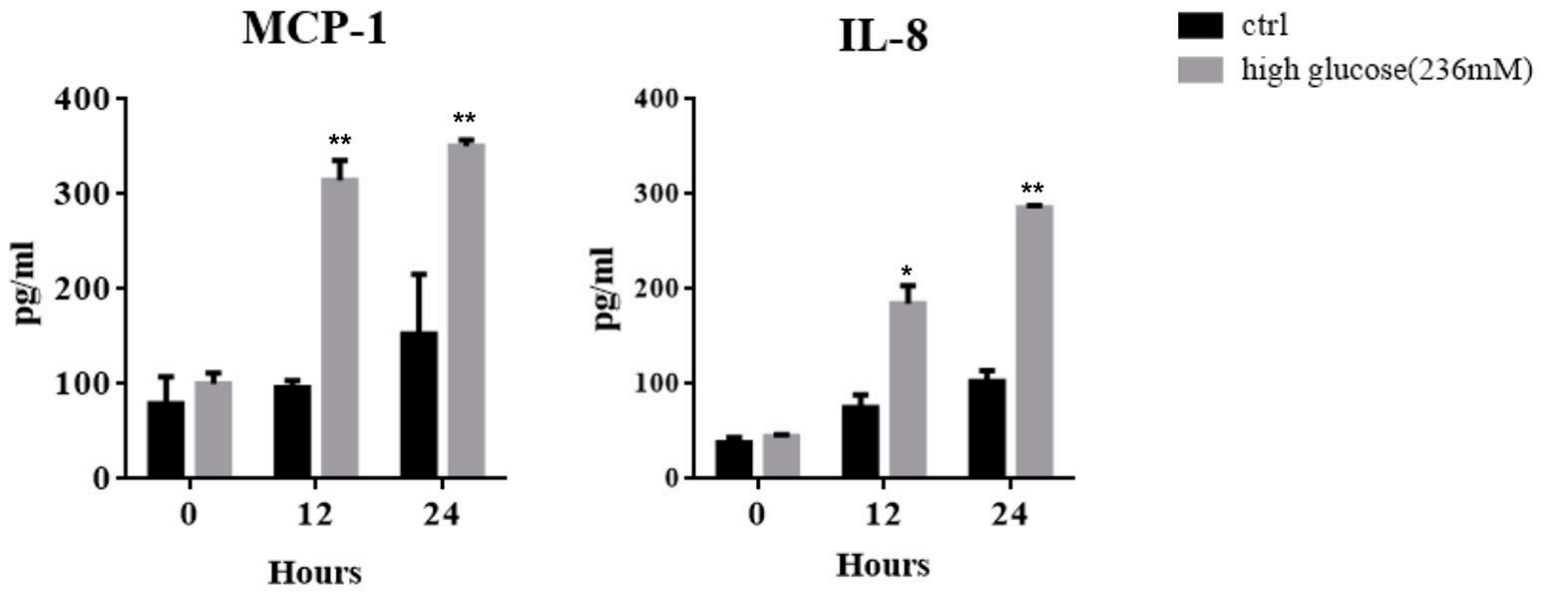
Mesothelial cells (MCs) were cultured for different periods in culture medium with or without high glucose (236 mM). ELISA was used to detect the concentrations of IL-8 and MCP-1 in the culture supernatant. Values are expressed as mean ± SEM (*n* = 3), ^*^p < 0.05 vs. control (ctrl); ^**^p < 0.01 vs. ctrl.

In keeping with clinical scenario, culture media glucose concentrations of 84, 138, and 236 mM were chosen to mimic the dialysates used for PD, which contain 1.5, 2.5, and 4.25% glucose. After treating MCs with high concentrations of glucose for different periods, increased concentrations of MCP-1 and IL-8 were found in the culture supernatant by ELISA (Figure 1), which is consistent with previous studies (Ha and Lee, 2000; Welten et al., 2003).

### High glucose promotes HMGB1 translocation and release from MCs

**Figure 2:**
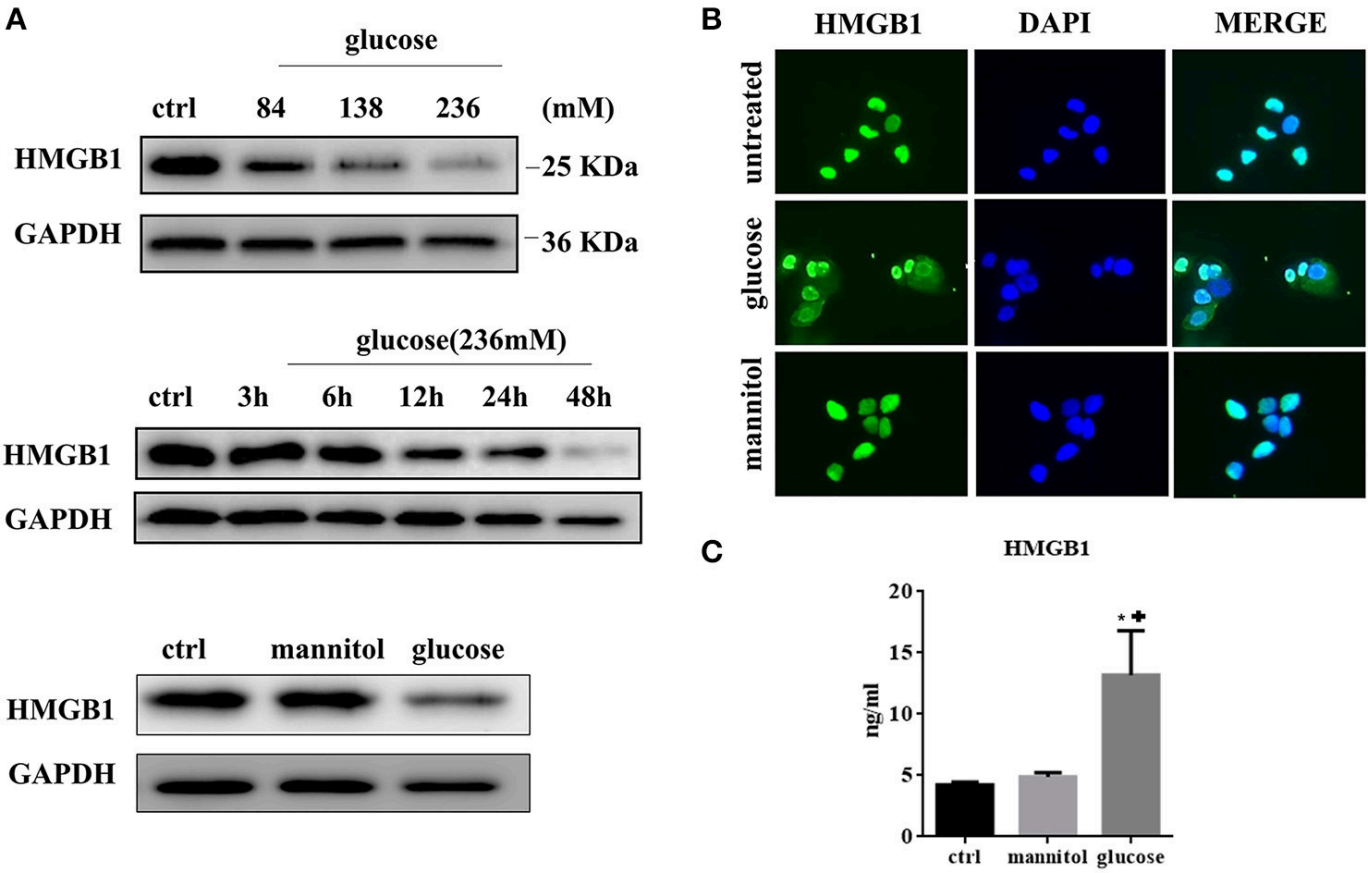
**(A)** HMGB1 expression was evaluated by western blotting assay in MCs treated with different doses of glucose for 48 h. (upper). MCs were treated with high concentration of glucose (236 mM) for differen time periods and analyzed for expression of HMGB1 protein by western blotting assay (middle). MCs were treated with glucose (236 mM) or mannitol at equimolar concentration for 48 h and intracellular HMGB1 was detected by western blotting assay (lower). **(B)** Immunofluorescent analysis of HMGB1 expression in MCs. Untreated MCs express HMGB1 in the nucleus, but partly lose HMGB1 nuclear expression, with cytoplasm accumulation after exposure to high glucose (236 mM for 48 h). **(C)** The levels of HMGB1 in the culture medium under different conditions (ctrl, 236 mM mannitol, and 236 mM glucose) for 48 h were tested by ELISA. Values are expressed as mean ± SEM (*n* = 3), ^*^P < 0.05 vs. ctrl; +*p* < 0.05 vs. mannitol.

We next tested the expression of HMGB1 in MCs after exposure to high glucose. MCs normally express high levels of HMGB1 in the nucleus. However, in the high-glucose condition, the HMGB1 content decreased in a time- and dose-dependent manner, as assessed by western blotting (Figure 2A). Immunofluorescent analysis showed an increase and decrease in cytoplasmic and nuclear fluorescence, respectively, under high glucose conditions, whereas the addition of mannitol to normal glucose did not result in the same effect (Figure 2B). Meanwhile, elevated HMGB1 levels were detected in culture supernatant. After 48 h of incubation HMGB1 level in culture supernatant was 4.22 ± 0.14 ng/ml in the control, 13.18 ± 2.10 ng/ml (*p* = 0.0130 vs. ctrl) in the 236 mM glucose, and 4.88 ± 0.21 ng/ml (*p* = 0.0582 vs. ctrl) in the 236 mM mannitol groups (Figure 2C). These data indicated that high concentrations of glucose induced HMGB1 secretion from the nucleus.

### Released HMGB1 stimulates MC production of MCP-1 and IL-8

**Figure 3:**
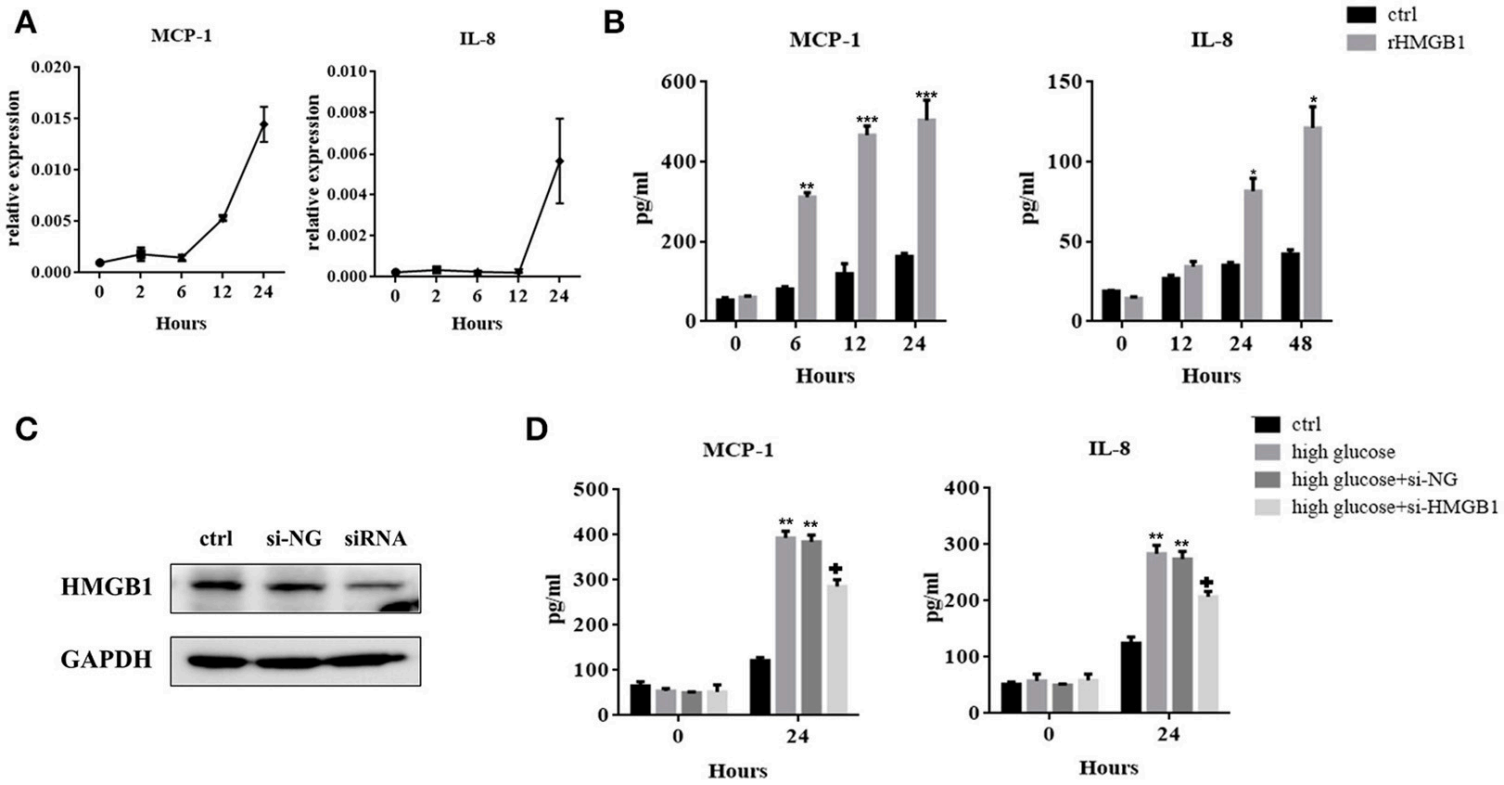
**(A)** MCs were incubated with recombinant HMGB1 (rHMGB1; 200 ng/mL) for different periods. Levels of MCP-1 and IL-8 mRNAs were analyzed by quantitative RT-PCR. **(B)** ELISA assay was used to detect the expression of IL-8 and MCP-1 in the culture supernatant after treatment with rHMGB1 (200 ng/mL) for different periods. Values are expressed as mean ± SEM (*n* = 3), ^***^p < 0.001 vs. ctrl; ^**^p < 0.01 vs. ctrl; ^*^p < 0.05 vs. ctrl. **(C)** Interference efficiency of HMGB1 siRNA in MCs was determined by western blot assay. **(D)** IL-8 and MCP-1 expression in si-HMGB1 group were significantly lower than in the high glucose (236 mM) and si-NC groups, as tested by ELISA. Values are expressed as mean ± SEM (*n* = 3), ^**^p < 0.01 vs. ctrl; +*p* < 0.05 vs. high glucose.

When secreted, HMGB1 evokes inflammatory responses via its interaction with cell surface-expressed receptors. We therefore investigated the role of HMGB1 in high-glucose-induced inflammatory response in MCs. Figures 3A,B showed that after incubation with rHMGB1, mRNA, and protein levels of MCP-1 and IL-8 increased in a time-dependent manner, but TNF-α was undetectable in this system (data not shown). Next, we silenced HMGB1 using siRNA. Western blotting assays were performed to measure HMGB1 expression after RNA interference (Figure 3C). Results showed that compared to the group with single exposure to high glucose or that was transfected with a negative control (si-NC), the expressions of IL-8 and MCP-1 decreased in the group transfected with HMGB1 siRNA, as assessed by ELISA (Figure 3D).

### HMGB1 has little effect on apoptosis of MCs induced by high glucose

**Figure 4:**
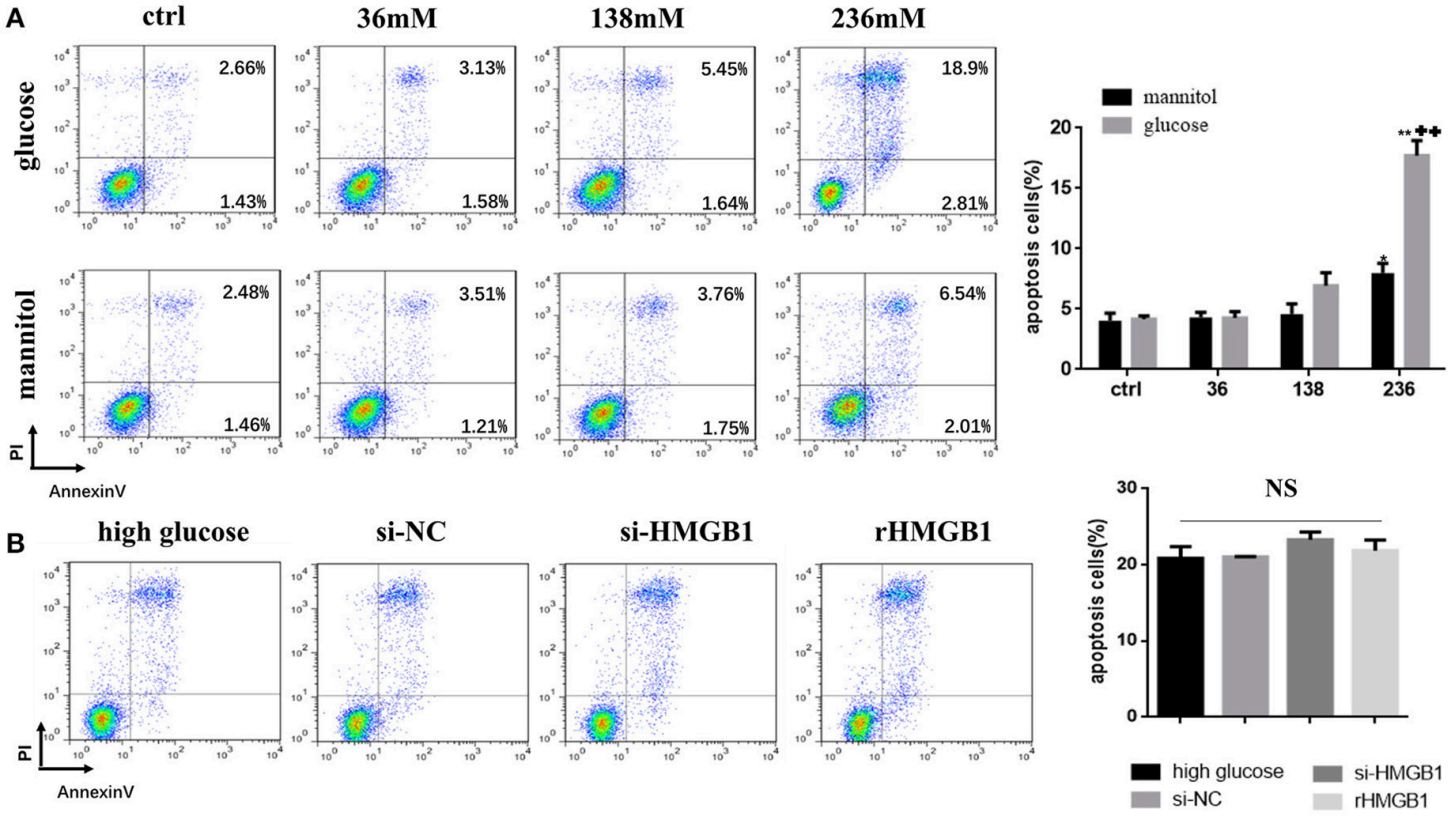
**(A)** Effect of high glucose or mannitol on MC apoptosis was measured by flow cytometry. Representative dot plots are shown, and percentage of cells positive for Annexin-V or positive for both Annexin-V and propidium iodide (PI) are represented as a histogram. Values are expressed as mean ± SEM (*n* = 3), ^*^p < 0.05 vs. ctrl; ^**^p < 0.01 vs. ctrl; ++*p* < 0.01 vs. 236 mM mannitol. **(B)** Apoptotic rate of high-glucose-treated MCs transfected with HMGB1 siRNA or incubated with rHMGB1 was tested by flow cytometry. NS means no significant difference.

MCs incubated in different glucose conditions and in mannitol at equimolar concentrations as osmotic controls were stained with Annexin V/propidium iodide for flow cytometry to distinguish and quantify the percentage of apoptotic cells. Results showed that compared to the normal glucose group, apoptotic rate in the high glucose group (236 mM for 24 h) markedly increased (Figure 4A), while a slight increase was observed in the mannitol group. We next tested the role of HMGB1 in high-glucose-induced apoptosis. Compared to single treatment with high glucose (236 mM) for 24 h, both intakes of rHMGB1 (200 ng/ml) and HMGB1silencing with siRNA in MCs had no significant effects on cellular apoptosis induced by high glucose, as determined by flow cytometry (Figure 4B). Thus, we speculated that HMGB1 did not participate in the high-glucose-induced apoptosis of MCs.

### Recombinant HMGB1 interacts with MCs through activation of MAPK signaling pathway

**Figure 5:**
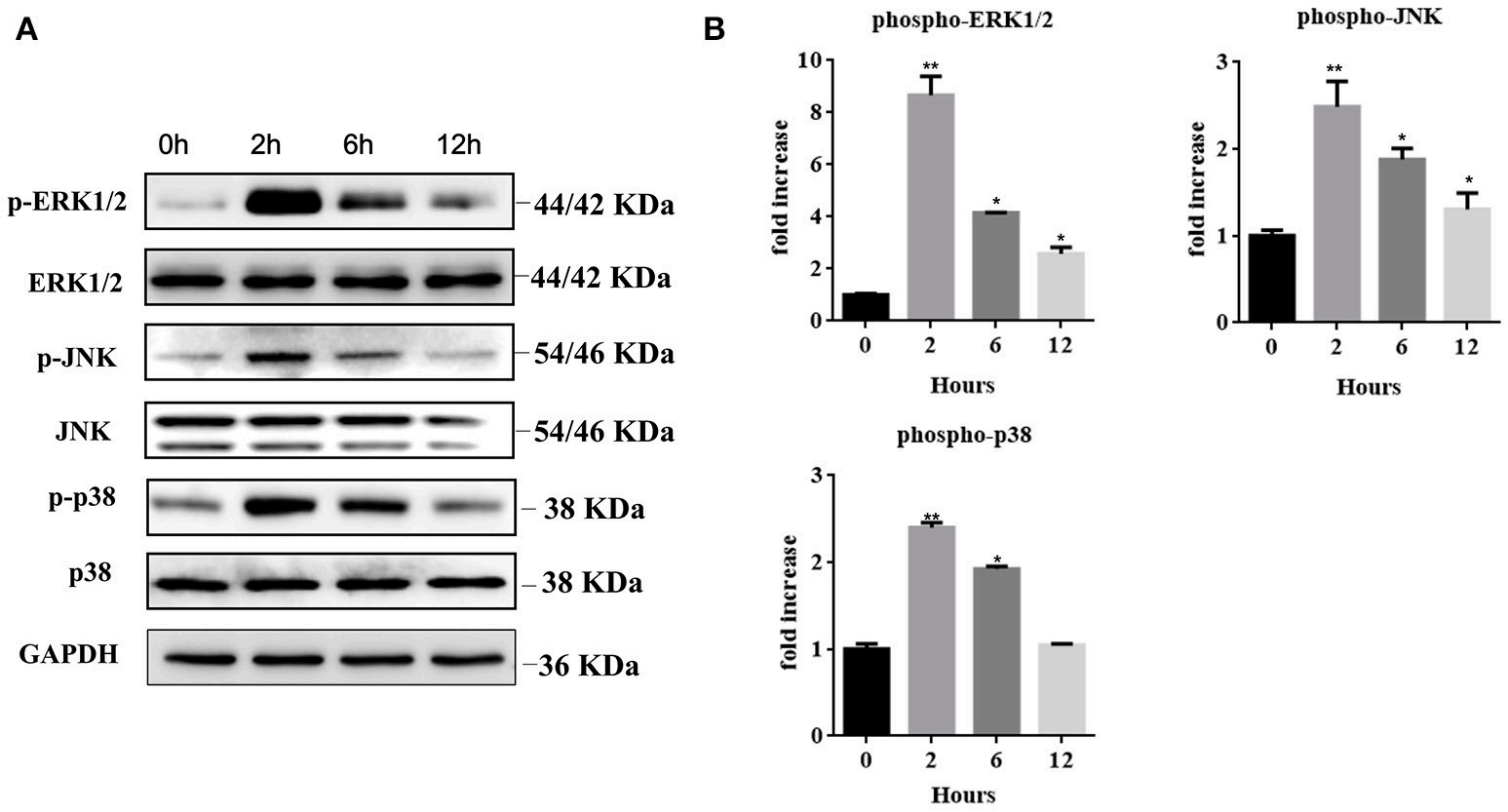
**(A)** Western blots of MCs treated with rHMGB1 (200 ng/ml) for the indicated times were probed with antibodies against the phosphorylated and common forms of ERK1/2, JNK, and p38. GAPDH was detected as loading control. **(B)** Quantification of Phospho-ERK1/2, JNK, and p38 was performed using Image J software. Values are expressed as mean ± SEM (*n* = 3), ^*^p < 0.05 vs. 0 h; ^**^p < 0.01 vs. 0 h.

**Figure 6:**
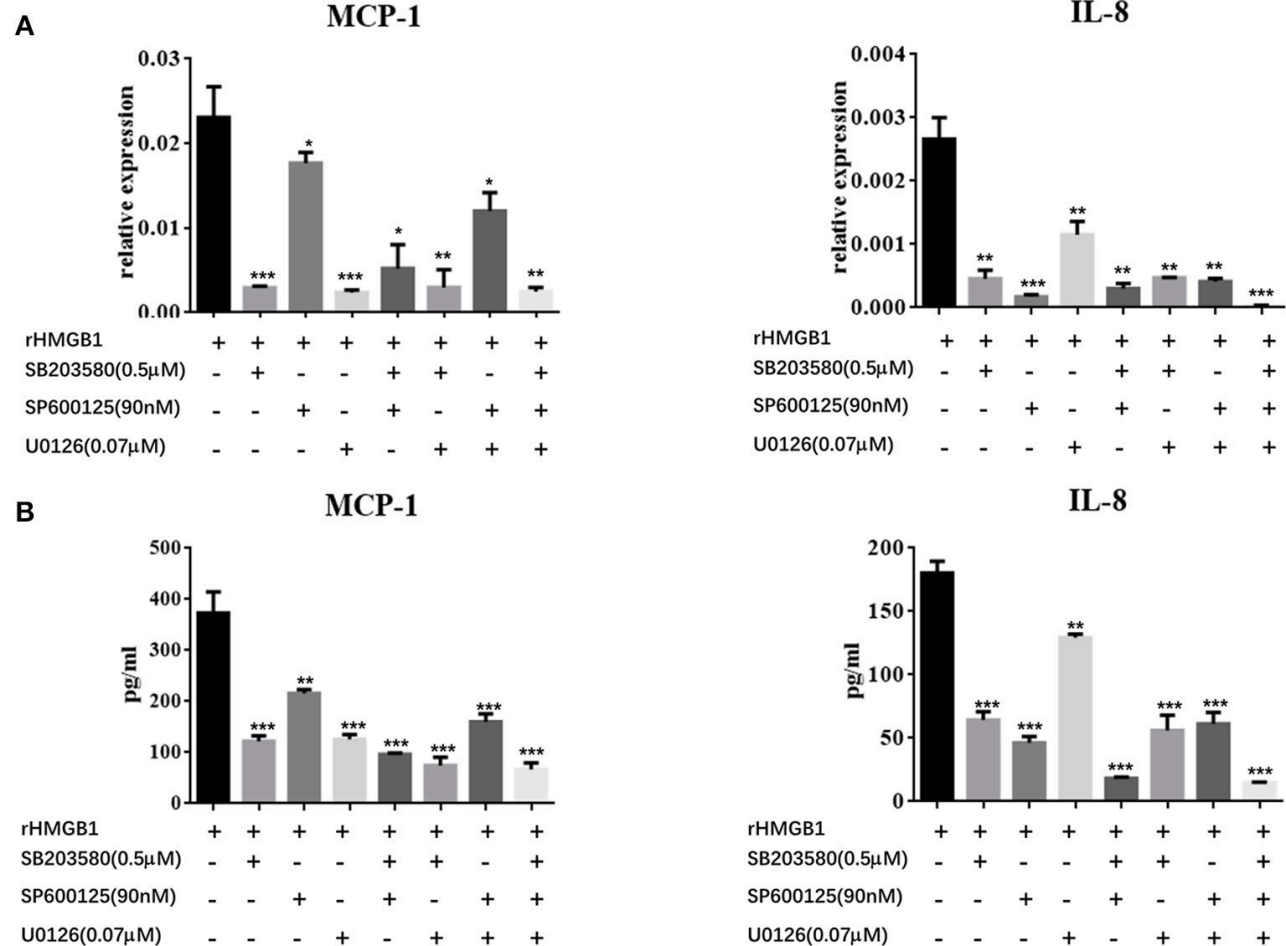
Effects of the specific p38 MAPK inhibitor, SB203580, ERK inhibitor, U0126, and JNK inhibitor, SP600125, on HMGB1-stimulated MCP-1 and IL-8 expression in MCs were determined by real-time PCR **(A)** and ELISA **(B)**. Values are expressed as mean ± SEM (*n* = 3), ^***^p < 0.001 vs. rHMGB1; ^**^p < 0.01 vs. rHMGB1; ^*^p < 0.05 vs. rHMGB1.

We further explored the regulatory mechanisms associated with MC activation by HMGB1. It has been reported that MAPK signaling pathways play crucial roles in the activation of HMGB1-meditated inflammation. We therefore analyzed three different MAP kinases (p38, ERK1/2, and JNK) by western blots in rHMGB1-treated MCs. Results showed that phosphorylated p38, ERK1/2, and JNK were all strongly increased (Figures 5A,B), suggesting that HMGB1 activated the p38, ERK1/2, and JNK signal pathways. We subsequently investigated the effects of specific p38 MAPK inhibitor, SB203580, ERK inhibitor, U0126, and JNK inhibitor, SP600125, on rHMGB1-mediated MCP-1 and IL-8 induction. Results indicated that SB203580, SP600125, and U0126 dramatically blocked the expression and secretion of rHMGB1-stimulated MCP-1 and IL-8 release as tested by real-time PCR and ELISA (Figures 6A,B). Taken together, HMGB1 promoted the expression of MCP-1 and IL-8, at least in part, via MAPK signaling pathways.

## Discussion

Persistent low-grade inflammation has been proven to be implicated in the pathogenesis of peritoneal fibrosis (Li et al., 2017). The currently used dialysates are glucose-based solutions with high concentrations of glucose. High glucose in PD solutions is a major cause of peritoneal inflammation and dysfunction (Cendoroglo et al., 1998; Sakamoto et al., 2005; Ranzinger et al., 2014). As a critical component of PM, MCs actively participate in the induction of inflammatory responses through secretion of MCP-1 and IL-8, which are two important chemotactic cytokines in the recruitment of leukocytes into the peritoneum (Topley et al., 1996; Li et al., 1998). We demonstrated that high glucose caused a time-dependent increase of MCP-1 and IL-8, which is similar to previous studies (Welten et al., 2003; Choi et al., 2017).

HMGB1 has been related to the occurrence and development of peritoneal dysfunction in several previous studies. Zhu et al. found that elevation of HMGB1 in serum correlated with micro-inflammatory state in continuous ambulatory PD patients (Zhu et al., 2011). Cao et al. reported that inhibition of HMGB1 expression elicited peritoneal protective effect in lipopolysaccharide (LPS)-induced peritoneal dysfunction in animal model (Cao et al., 2013). These findings uncovered the pathogenic relevance of HMGB1 in peritoneal inflammation. In this study, we demonstrated the ability of high concentrations of glucose to induce the release of HMGB1 in MCs, and its role in MCP-1 and IL-8 production. It is well-accepted that two major pathways of HMGB1 release occur during injury; one is active secretion by stimulated cells and the other is passive release by necrotic cells. Cytoplasmic translocation of HMGB1 induced by high glucose has been reported in many cells including retinal pericytes and vascular smooth muscle cells (Wang et al., 2013; Kim et al., 2016). In our study, similar effect was observed in MCs. Figure 2 showed that HMGB1 content was depleted in a time- and dose-dependent manner in MCs after exposure to high glucose. High glucose resulted in increased MC cytosolic expression and subsequent extracellular levels of HMGB1. Long-term exposure of MCs to high glucose condition could induce MC injury due to hyperosmotic pressure and metabolic effect (Breborowicz et al., 1992). Thus, it is possible that MC necrosis induced by hyperosmotic pressure and metabolic effect serve as additional contributors to the extracellular HMGB1 accumulation. On the one hand, as tested by ELISA, we found a slight and sharp elevation of extracellular HMGB1 in mannitol-treated and high-glucose-treated groups, respectively, indicating that extracellular HMGB1 accumulation mainly resulted from high glucose and partly by osmotic pressure. On the other hand, passive release due to metabolic effect should be considered as another mechanism involved in extracellular HMGB1 accumulation as demonstrated by flow cytometry, showing that high glucose strongly increased apoptotic rate of MCs compared to normal glucose and mannitol. Taken together, we concluded that high glucose was an important regulator of subcellular distribution of HMGB1 in MCs. Once released, HMGB1 can directly promote the release of various inflammatory cytokines such as MCP-1, IL-8, and TNF-α by pattern recognition receptor (PRR)-expressing cells (Fiuza et al., 2003). We also confirmed that secreted HMGB1 acted on MCs to promote the release of cytokines. By ELISA, we found that rHMGB1 induced increased expressions of MCP-1 and IL-8, but not TNF-α. In human microvascular endothelial cells, the proinflammatory activity of HMGB1 was amplified by local TNF production (Fiuza et al., 2003). However, different from endothelial cells, rHMGB1-treated MCs strongly elevated the levels of IL-8 and MCP-1, but failed to induce the production of TNF-α, suggesting that HMGB1-induced chemotactic cytokine secretion was TNF independent. It must be noted that the proinflammatory function of HMGB1 relies on its binding to PRRs. Choi et al. have reported that TLR4 was partly involved in the high-glucose-induced MCP-1 expression by MCs (Choi et al., 2017). In the present study, we did not explore the types of receptors engaged in the interaction between HMGB1 and MCs, which should be studied in future experiments.

A previous study inhibited HMGB1 by pretreatment with glycyrrhizin, a direct HMGB1 antagonist, to confirm its protective effect on peritoneal function (Cao et al., 2013). RNA interference is a powerful tool for post-transcriptional gene silencing. Silencing HMGB1 with siRNA has been widely used in many experiments (Huang et al., 2015; Ni et al., 2015). We knocked down HMGB1 in MCs and found that silencing HMGB1 with siRNA moderately reduced MCP-1 and IL-8 release induced by high concentrations of glucose, which confirmed the proinflammatory effect of HMGB1 on MCs.

Among all the involved pro-inflammatory pathways, MAPK signaling pathways play crucial roles in inflammatory responses. MAPK family members (ERK, JNK, and p38) were reported to induce cytokine secretion in response to HMGB1 stimulation in many cells, including mesenchymal stem cells (Feng et al., 2016). As hypothesized, our study manifested similar effects in MCs. HMGB1 directly activated p38, ERK1/2, and JNK in MCs. Inhibition of ERK1/2, p38, and JNK with specific inhibitors reduced the expression of MCP-1 and IL-8, suggesting the involvement of MAPK pathways in HMGB1 induced MCP-1 and IL-8 secretion by MCs.

High glucose-induced apoptosis is an important factor contributing to the loss of MCs during PD. Consistent with previous studies, we also demonstrated a dose dependent increase of apoptosis in MCs under high glucose conditions. The effect of HMGB1 on apoptosis is complicated. Studies in cancer cells show that HMGB1 is a regulator of the balance between autophagy and apoptosis. Released HMGB1 triggers autophagy, which limits programmed apoptotic cell death. Diminished HMGB1 by siRNA transfection caused accelerated apoptosis (Tang et al., 2010). In contrast, HMGB1 knockdown by siRNA inhibited apoptosis in human retinal endothelial cells induced by high glucose (Jiang and Chen, 2017). In the present study, we demonstrated that pretreatment with HMGB1 siRNA apparently failed to influence MC apoptosis in high glucose condition, implying the minor role of HMGB1 on high-glucose-mediated apoptosis of MC.

## Conclusion

**Figure 7:**
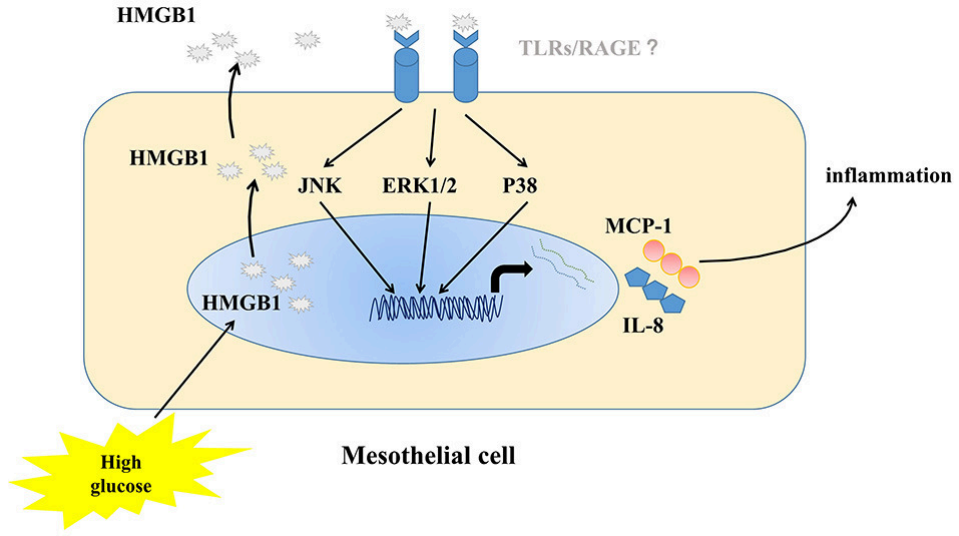
Schematic model of the proposed signal pathways of inflammation induced by high glucose in MC.

On the basis of these observations, we conclude that high concentrations of glucose could stimulate cytoplasmic translocation and release of HMGB1 to act in an autocrine manner on MCs to induce up-regulated expression of two important chemotactic factors (IL-8 and MCP-1) to amplify the inflammatory reaction. In addition, HMGB1-meditated inflammation was mediated by MAPK pathway activation. Figure 7 showed a schematic model of the proposed signal pathways of inflammation induced by high glucose in MC. Endogenous HMGB1 has little effect on high-glucose-induced apoptosis of MCs. These results demonstrate a new mechanism of high-glucose-induced inflammatory effects on MCs, which provide useful evidence to our understanding of how high concentrations of glucose, could exacerbate MC disruption and microenvironmental changes in peritoneal cavity.

## Author contributions

YC, ZZ, YW, and YL performed experiments; YC, YL, RH, JL, and XY wrote the manuscript; YC, YL, and JL contributed to manuscript preparation. All authors read and approved the final version of this manuscript for submission.

### Conflict of interest statement

The authors declare that the research was conducted in the absence of any commercial or financial relationships that could be construed as a potential conflict of interest.
